# Identification of the pyridoxal 5′‐phosphate allosteric site in human pyridox(am)ine 5′‐phosphate oxidase

**DOI:** 10.1002/pro.4900

**Published:** 2024-01-23

**Authors:** Anna Barile, Claudio Graziani, Lorenzo Antonelli, Alessia Parroni, Annarita Fiorillo, Martino Luigi di Salvo, Andrea Ilari, Alessandra Giorgi, Serena Rosignoli, Alessandro Paiardini, Roberto Contestabile, Angela Tramonti

**Affiliations:** ^1^ Istituto di Biologia e Patologia Molecolari Consiglio Nazionale delle Ricerche Rome Italy; ^2^ Sapienza Università di Roma Istituto Pasteur Italia‐Fondazione Cenci Bolognetti Rome Italy; ^3^ Dipartimento di Scienze Biochimiche “A. Rossi Fanelli” Sapienza Università di Roma Rome Italy

**Keywords:** allosteric inhibition, molecular docking, PNPO, vitamin B_6_ salvage pathway, x‐ray crystallography

## Abstract

Adequate levels of pyridoxal 5′‐phosphate (PLP), the catalytically active form of vitamin B_6_, and its proper distribution in the body are essential for human health. The PLP recycling pathway plays a crucial role in these processes and its defects cause severe neurological diseases. The enzyme pyridox(am)ine 5′‐phosphate oxidase (PNPO), whose catalytic action yields PLP, is one of the key players in this pathway. Mutations in the gene encoding PNPO are responsible for a severe form of neonatal epilepsy. Recently, PNPO has also been described as a potential target for chemotherapeutic agents. Our laboratory has highlighted the crucial role of PNPO in the regulation of PLP levels in the cell, which occurs via a feedback inhibition mechanism of the enzyme, exerted by binding of PLP at an allosteric site. Through docking analyses and site‐directed mutagenesis experiments, here we identified the allosteric PLP binding site of human PNPO. This site is located in the same protein region as the allosteric site we previously identified in the *Escherichia coli* enzyme homologue. However, the identity and arrangement of the amino acid residues involved in PLP binding are completely different and resemble those of the active site of PLP‐dependent enzymes. The identification of the PLP allosteric site of human PNPO paves the way for the rational design of enzyme inhibitors as potential anti‐cancer compounds.

## 1 INTRODUCTION

Vitamin B_6_ is an ensemble of six substituted pyridine compounds or vitamers: pyridoxine (PN), pyridoxal (PL), pyridoxamine (PM), and their related 5′‐phosphate derivatives. Among these, pyridoxal 5′‐phosphate (PLP) acts as a cofactor for various enzymes involved in many metabolic pathways (synthesis, transformation and degradation of amines and amino acids, supply of one carbon units, transsulfuration, synthesis of tetrapyrrolic compounds and polyamines, biosynthesis and degradation of neurotransmitters) (Percudani & Peracchi, 2003). In humans, the different B_6_ vitamers, introduced with food, are interconverted through a salvage pathway which involves, besides kinases and phosphatases, the enzyme pyridoxine 5′‐phosphate (PNP)/pyridoxamine 5′‐phosphate (PMP) oxidase (PNPO) (di Salvo et al., 2011, 2012). This flavin mononucleotide (FMN)‐dependent enzyme, which produces PLP from PNP or PMP, plays an active role in vitamin B_6_ homeostasis. Its key function as PLP supplier is demonstrated by the observation that mutations in the *PNPO* gene are responsible for an autosomal recessive inborn error of metabolism, namely PNPO deficiency (PNPOD; OMIM: 610090). Patients affected by PNPOD show severe neonatal‐onset seizures, which can be treated mainly with PLP or in some cases with PN (Wilson et al., 2019). In microorganisms, like *Escherichia coli*, which synthesize PLP through the so‐called de novo DXP‐dependent pathway, the PNPO reaction represents the last irreplaceable step of PLP biosynthesis (Tramonti et al., 2021). In both human and *E. coli* PNPO, PLP works as a regulator of its own production because it binds at an allosteric site of the enzyme, affecting the binding of substrate at the active site and inhibiting the catalytic activity (Barile et al., 2019, 2021). This shows that a cross‐talk between the allosteric site and the active site is present. In the *E. coli* enzyme, functional and structural data have demonstrated that PLP binds with high affinity to an allosteric site made by three arginine residues that form an “arginine‐cage” motif (Barile et al., 2021). When located in this site, PLP induces a structural rearrangement: in particular, it uncoils a short helix, perturbing the active site and displacing an important catalytic residue, Arg197, involved in binding and correct positioning of the substrate (Barile et al., 2021). As a consequence, the enzyme with PLP bound at the allosteric site is completely inactive (Barile et al., 2019). In the human PNPO, the coupling between the allosteric site and the active site is weaker, and PLP can bind simultaneously at the allosteric site and at the active site, forming a complex in which each subunit binds two PLP molecules. Moreover, the enzyme with PLP bound at the allosteric site maintains a partial catalytic activity (Barile et al., 2020). To date, the actual location of the allosteric PLP binding site in the structure of the human PNPO is unknown. Multiple sequence alignments (MSAs) show the lack of an arginine‐cage motif in the human enzyme. An initial docking analysis performed in this study indicated the possible location of the allosteric PLP binding site, which was then verified by the characterization of site‐directed variant enzymes. Moreover, comparison of new crystal structures of PNPO with others previously solved, together with the observation of a reproducible proteolysis of the recombinant enzyme, suggest a marked flexibility of the N‐terminal portion of human PNPO. The identification of the PLP allosteric site in human PNPO could be important considering that this enzyme represents an interesting target of chemotherapy agents (Chen et al., 2017; Ren et al., 2019; Zhang et al., 2017). Several studies have highlighted the great importance of PLP in cancer progression by sustaining the metabolic requirements of highly proliferating cells. In fact, PLP supplementation in many cancer models promotes tumor progression (Tryfiates et al., 1978). Moreover, PNPO is overexpressed in some tumors (Chen et al., 2020; Ren et al., 2019; Zhang et al., 2017, 2021), whereas its silencing counteracts tumor progression (Ren et al., 2019; Zhang et al., 2017). A well‐modulated inhibition of PNPO could therefore represent a possible anti‐cancer therapy. The design of specific PNPO inhibitors cannot be achieved without detailed knowledge of the PLP allosteric binding site.

## 2 RESULTS

### 2.1 Molecular docking experiments on human PNPO structures identified a putative PLP binding site

In order to identify the actual location of the allosteric PLP binding site, structural bioinformatics and docking studies were carried out. In the *E. coli* enzyme, PLP binds strongly to an “arginine‐cage” motif (Barile et al., 2021). Before its identification, this site could potentially be predicted through the analysis of the PNPO crystal structure from *E. coli* (PDB: 1G78; Safo et al., 2001), in which a phosphate ion from the crystallization buffer remained bound to the arginine cage. Therefore, following a similar strategy, our initial aim was to: (1) identify superficial pockets in the human PNPO protein structures (see Section 4 for details) with evolutionarily conserved residues, binding phosphate ions; (2) docking of PLP into the identified pockets, in order to assess its potential binding mode (Figure S1). Through this approach, we identified three potential binding sites, which were confirmed by docking studies (hereinafter sites I–III).

In site I, PLP is hypothesized to bind in a deep conserved cleft where the highly conserved Asn112 is nestled at the depths of a pocket, and is envisaged to contribute to the stabilization of the pyridine moiety via interaction with the N‐atom of the ring. Additionally, His173 is proposed to engage in a stacking interaction with the PLP ring, enhancing stabilization. Another noteworthy interaction emerges between Glu114 and Ser176. Their pairing offers the intriguing possibility of supporting the phosphate group (Figure S1). In site II, notably, Lys100 assumes a strategic position to potentially bind PLP covalently. Moving along the proposed interactions in the docking simulation, Ser160 forms a hydrogen bond with the PLP aldehydic functional group. Finally, Glu153 and Arg108 are at interaction distance with the phosphate moiety of PLP (Figure S1).

**FIGURE 1:**
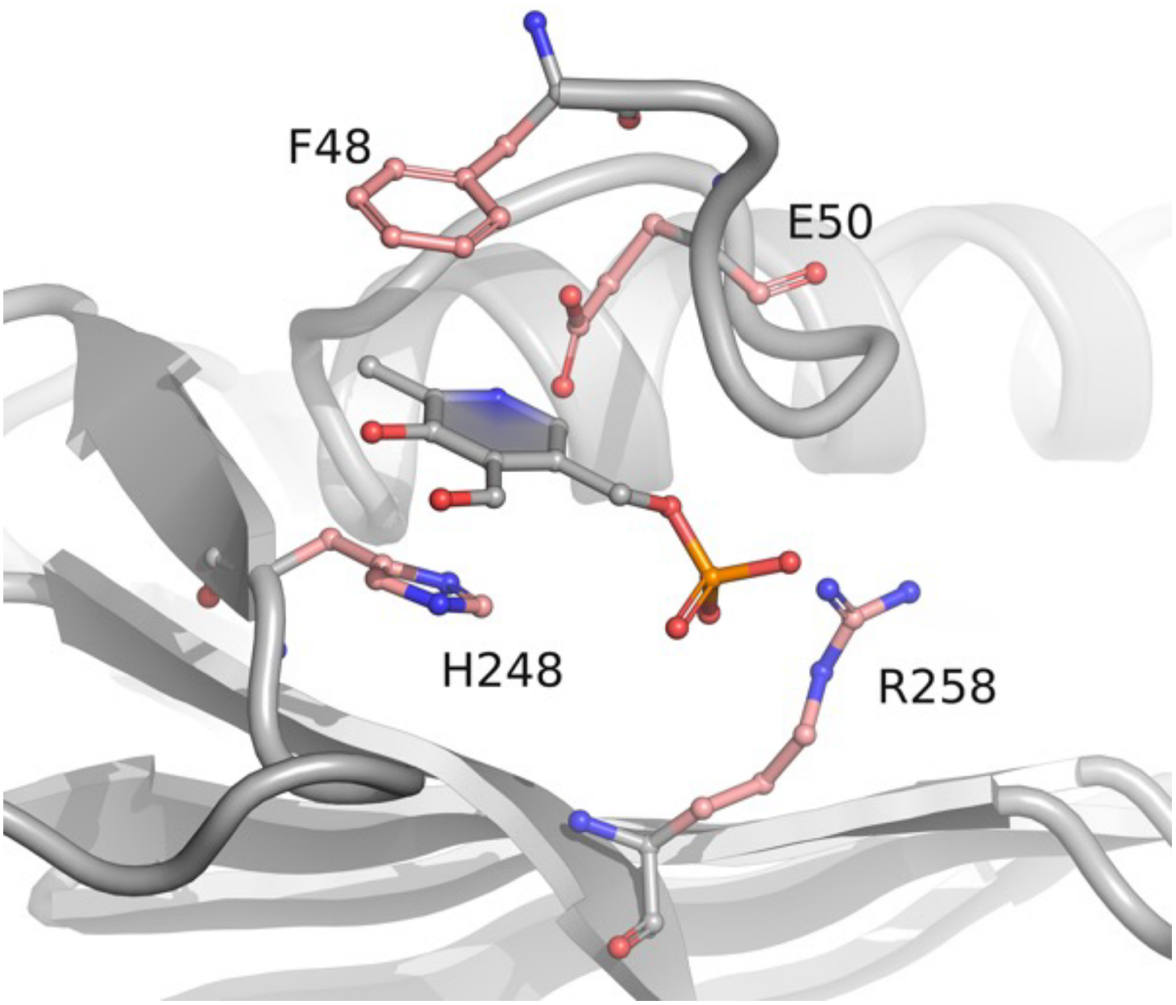
Docking of PLP at a conserved cleft of human PNPO. PNPO is shown as gray ribbons. PLP is represented as gray balls‐and‐sticks model. Site‐directed mutagenesis was applied to the residues highlighted in pink.

Of the three identified pockets, site III seemed the most promising and captured our attention, for a number of reasons. First and most notably, this site is located in the same position of the PLP allosteric site found in *E. coli* PNPO. Moreover, the PLP ring is nicely double‐stacked between the aromatic residues Phe48 and His248. Finally, as observed in many PLP‐dependent enzymes, a glutamate residue (Glu50) interacts with the N‐atom of the pyridine ring, while an arginine (Arg258) is involved in phosphate stabilization (Figure 1).

### 2.2 Catalytic properties of variants that hit the putative allosteric PLP binding site

To verify the actual involvement of the putative PLP binding site III identified through docking analyses in the allosteric mechanism of human PNPO, three variant forms of the enzyme were produced through site‐directed mutagenesis and characterized: a single (F48A), a triple (F48A/E50L/R258L) and a quadruple (F48A/E50L/H248N/R258L) variant.

The catalytic properties of these three variants were analyzed in 50 mM Tris–HCl buffer, pH 7.6, at 37°C, using both PNP and PMP as substrate (Figures S2 and S3). In these conditions, the PLP product forms an aldimine linkage with Tris and does not accumulate in the solvent. As a consequence, PLP does not inhibit the enzyme activity by binding at the allosteric site. The obtained kinetic parameters are shown in Table 1. All variations affected K_M_ for PNP, that increased up to 10‐fold with respect to that of the wild type enzyme. At the same time, *k*_CAT_ values only moderately increased. When PMP was used as substrate, the wild type enzyme showed a larger *K*_M_ for this substrate, which was 25‐fold higher than that for PNP. This observation is in contrast with what reported in a previously published work, in which *K*_M_ for PNP and *K*_M_ for PMP were similar (Musayev et al., 2003). The three variants showed a 1.5‐fold larger K_M_ for PMP with respect to the wild type enzyme, whereas the *k*_CAT_ was substantially unaffected. It is worth noticing that, with respect to the single F48A variant, the introduction of two or three further variations did not lead to large effects on the kinetic parameters. On the other hand, while both the F48A and the triple variants showed a far‐UV CD spectrum superimposable to that of the wild type, the quadruple variant showed a more pronounced negative Cotton effect (Figure S4).

**TABLE 1:** Kinetic parameters and PLP dissociation constant values for the wild type and variant forms of human PNPO.

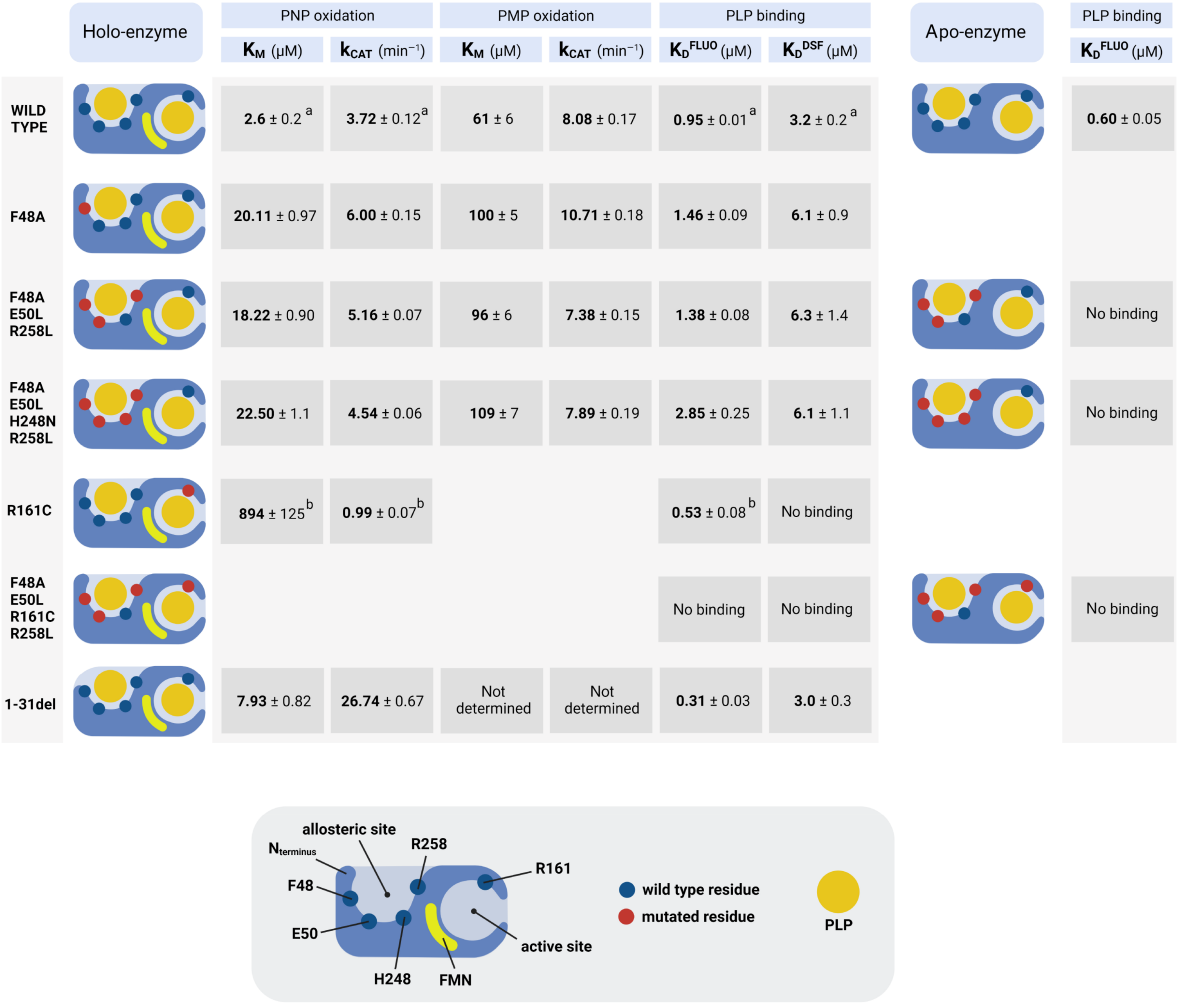

*Note*: A single subunit of PNPO is represented in cartoon style, with the dots indicating the position of the wild type (blue dots) or mutated (red dots) residues as described in the text. *K*_M_ and *k*_CAT_ values were measured by using either PNP and PMP as substrates. *K*_D_^FLUO^ is the dissociation constant value obtained by FMN fluorescence changes, while *K*_D_^DSF^ refers to the dissociation constant measured by DSF assays, as indicated in the text.

^a^From Barile et al. (2020).

^b^From Barile et al. (2021).

### 2.3 PLP binding to PNPO variants

**FIGURE 2:**
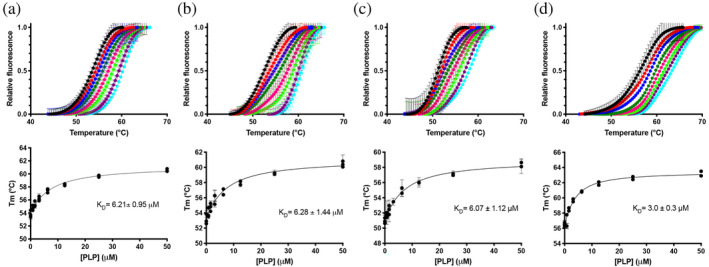
DSF measurements with F48A (a), F48A/E50L/R258L (b), F48A/E50L/H248N/R258L (c), and 1‐31del PNPO variants in the presence of different PLP concentrations. Upper panels: Fluorescence change is expressed as fractional variation as a function of temperature. The experiment was carried out using 2 μM enzyme and different PLP concentrations (0, 0.78, 1.56, 3.13, 6.25, 12.5, 25, and 50 μM). Thermal denaturation data are fitted to the Boltzmann equation to obtain melting temperatures. Each curve is the average of three independent experiments with standard error bars. Lower panels: Saturation curves obtained by plotting the melting temperatures as a function of the PLP concentration.

**FIGURE 3:**
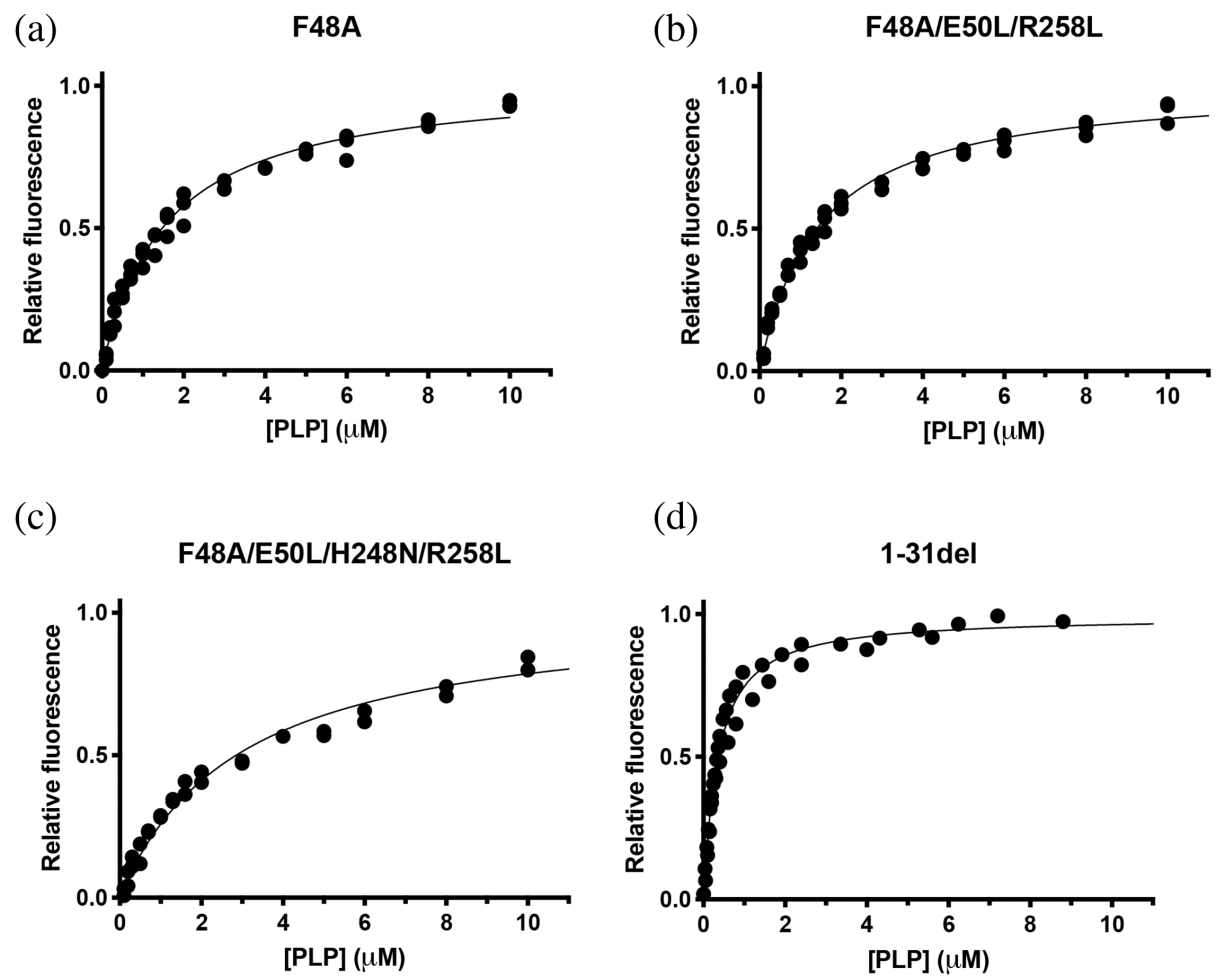
Analysis of PLP binding equilibrium of PNPO variants. PLP binding curves obtained with 100 nM PNPO variants. Reported data are the average ± standard deviation of three independent measurements. Data were fitted as described in Barile et al. (2020) and the resulting K_D_ values were reported in Table 1.

**FIGURE 4:**
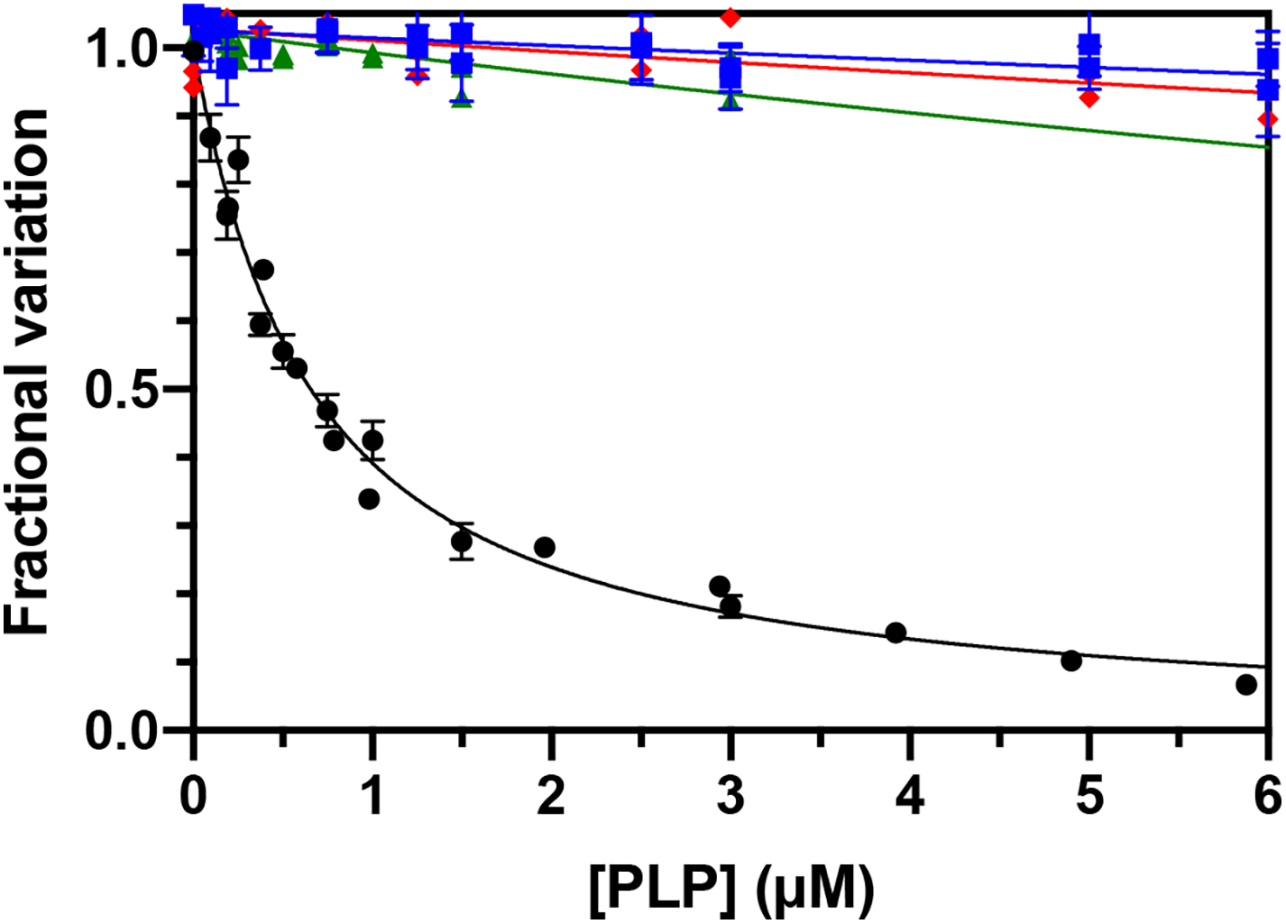
Analysis of PLP binding equilibrium of apo wt and variant forms of PNPO. PLP binding curves obtained with 100 nM apo‐PNPO wt (●), F48A/E50L/R258L (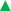), F48A/E50L/H248N/R258L (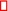), and F48A/E50L/R161C/R258L (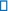). Reported data are the average ± standard deviation of three independent measurements. Data were fitted to Equation (2) and the resulting K_D_ values were reported in Table 1.

PLP binding to PNPO can be detected and quantitatively measured using differential scanning fluorimetry (DSF) and also by measuring FMN fluorescence changes. We had previously attributed DSF changes to PLP binding at the active site, while FMN fluorescence changes were attributed to PLP binding at the allosteric site (Barile et al., 2019, 2020). Previous experiments carried out with the PNPO R161C variant, affecting a residue that is essential for substrate binding and catalysis (and therefore also for PLP binding at the active site), confirms that DSF measurements only report PLP binding at the active site (Barile et al., 2021; Table 1). The dissociation constant values obtained with DSF are almost the same for the three variants and doubled with respect to the wild type enzyme (Figure 2, Table 1). This behavior reflects the decrease of affinity of these variants for the substrates compared to the wild type PNPO. We expected to observe no FMN fluorescence changes upon titration of the variants with PLP. However, this was not the case, and the dissociation constant values obtained by observing the FMN fluorescence emission were only up to threefold larger in the variants with respect to wild type PNPO (Figure 3). This suggests that either: (1) site III is not the PLP allosteric site, or (2) PLP binding at the active site may affect FMN fluorescence. The crystal structure of PNPO in complex with PLP showed that the co‐crystallized PLP is located at the active site with the pyridine ring stacked parallel against the pyrazine and pyrimidine portions of the FMN isoalloxazine ring, with extensive van der Waals contacts between the two (Musayev et al., 2003). To check the second possibility, we measured intrinsic fluorescence changes of wild type and variant apo‐forms (i.e., without FMN) upon PLP titration. The wild type apo‐form, analyzed by following the decrease in emitted fluorescence at 335–345 nm upon excitation at 280 nm, gave a *K*_D_ of 0.60 ± 0.05 μM. Interestingly with the triple (F48A/E50L/R258L) and the quadruple (F48A/E50L/H248N/R258L) variant apo‐forms, no change in fluorescence was observed upon PLP titration (Figure 4). These results strongly suggest that site III actually corresponds to the PLP allosteric site. Furthermore, we introduced the R161C variation into the triple (F48A/E50L/R258L) variant, obtaining a second quadruple variant (F48A/E50L/R161C/R258L). This variant enzyme, despite being purified by following the same method used for the wild type and the other variants, contained a low amount of FMN (only 34.7 ± 0.5% saturation with respect to the protein subunit). Both 35%‐saturated and apo‐form of this quadruple variant were titrated with PLP by exciting at 280 nm, and no variation in the emitted fluorescence was observed (Figure 4). These results confirmed that the four variations eliminated PLP binding at both the active and allosteric sites.

### 2.4 Analysis of inhibition kinetics

**SCHEME 1:**
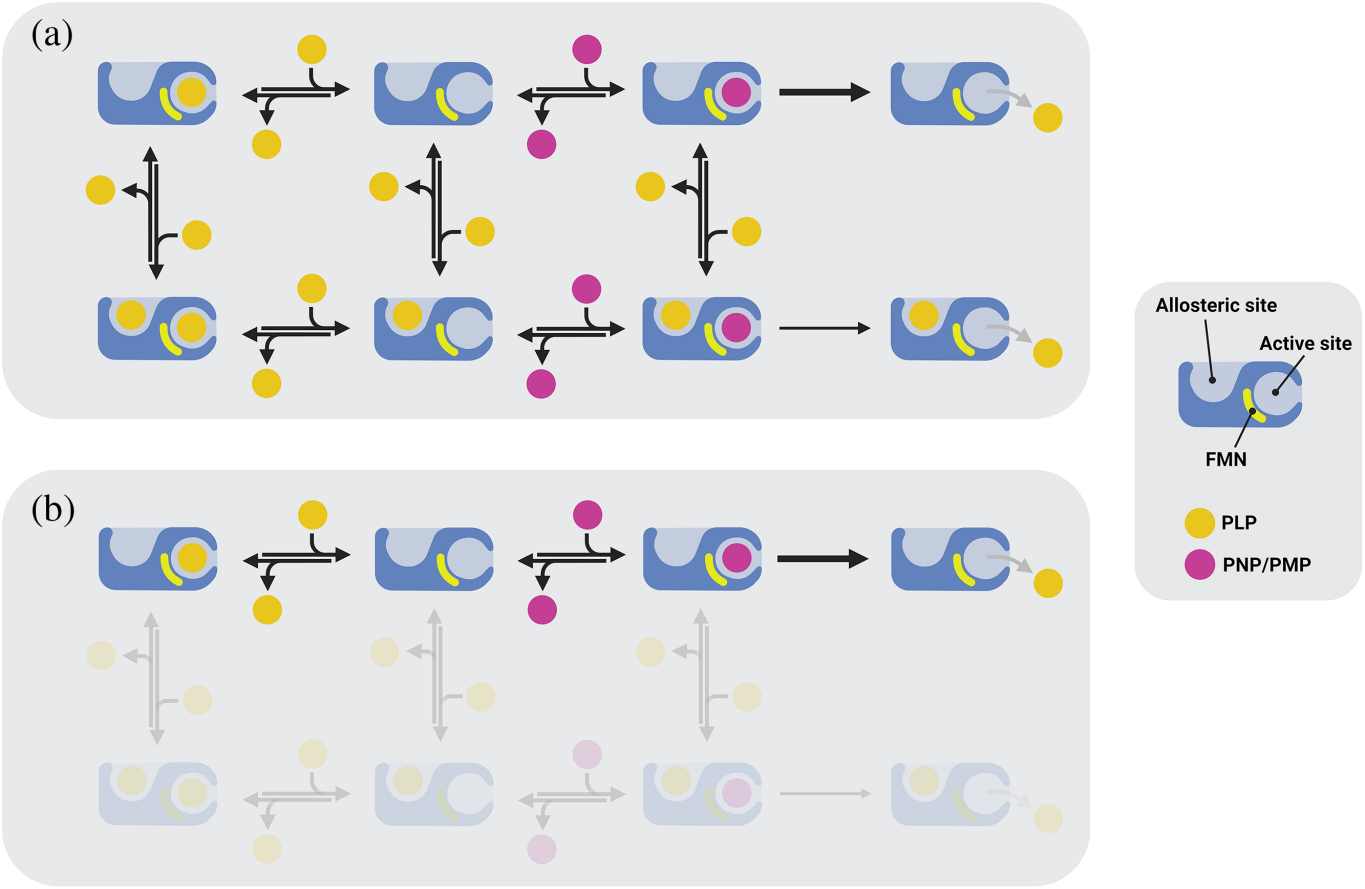
Simplified inhibition model representing PLP and PNP binding to PNPO. (a) Inhibition model for the wild type enzyme. The free enzyme is able to bind both the substrate (PNP, represented as a purple circle) and the product (PLP, represented as a yellow circle). PLP can bind either at the active (orthosteric) site or at the allosteric site, or at both of them. According to this model, PLP binding at the active site forms a dead‐end complex, unable to accept the substrate and perform the reaction. When PLP binds only at the allosteric site, however, it can oxidize PNP with a reduced efficiency. (b) Inhibition model for the F48A/E50L/H248N/R258L variant. This form cannot interact with PLP at the allosteric binding pocket, and it does not show any allosteric inhibition. The competitive inhibition, however, is retained.

**FIGURE 5:**
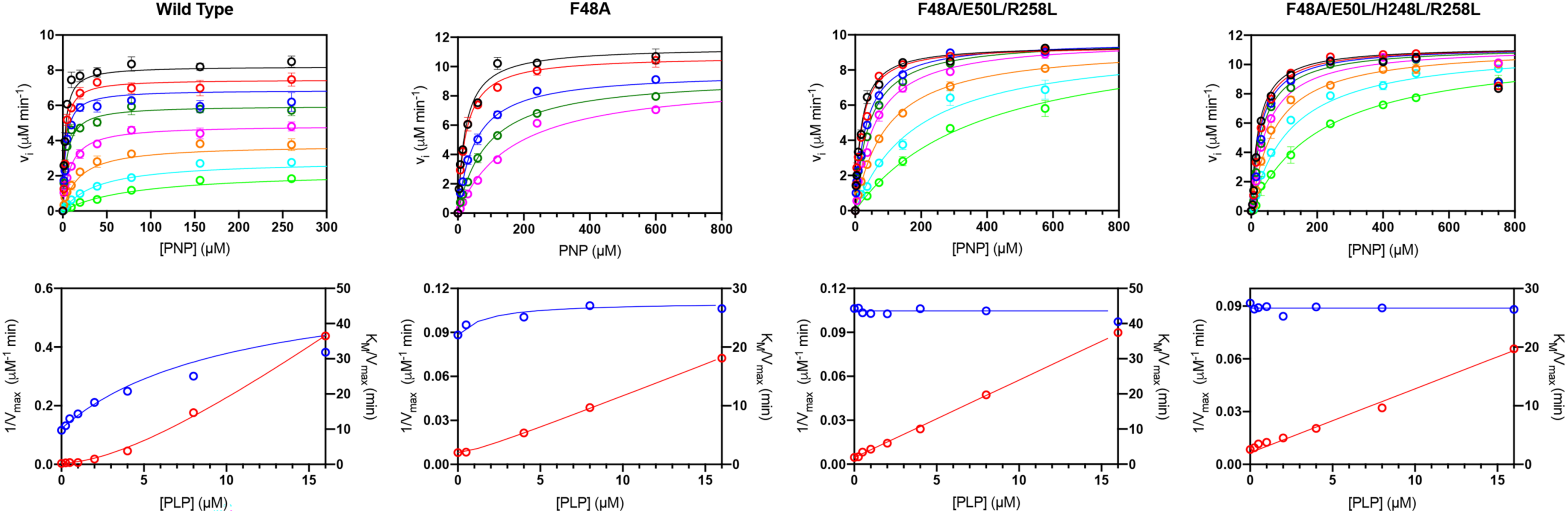
Characterization of PLP inhibition. Upper panels: The initial velocity of the reaction was measured with 2 μM wild type (a), and the F48A (b), F48A/E50L/R258L (c), and F48A/E50L/H248N/R258L (d) PNPO variants, varying PNP concentration while keeping exogenous PLP fixed at different concentrations (0, 0.25, 0.5, 1, 2, 4, 8, and 16 μM). Data are the average ± standard deviation of three independent measurements. The resulting saturation curves were fitted to the Michaelis–Menten equation, obtaining apparent *V*_max_ and *K*_M_ values at all PLP concentrations. Lower panels: Fitting of 1/*V*_max_ (blue symbols) and *K*_M_/*V*_max_ (red symbols), obtained from the fitting of data shown in upper panels, using an increasing hyperbolic equation and a parabolic equation, respectively.

Our previous studies characterized the allosteric properties of wild type human PNPO, revealing a parabolic inhibition mediated by PLP, as elucidated in Scheme 1a (Barile et al., 2020). This is a general inhibition mechanism that takes into account the possibility of PLP binding to both the active and allosteric sites. PLP binding at the active site gives a complete, competitive inhibition, whereas PLP binding at the allosteric site yields a substrate‐enzyme‐PLP ternary complex that is still catalytically active, although *k*_CAT_ is reduced by a coefficient whose value is around 0.15. A thorough inhibition kinetic characterization was carried out with the wild type, F48A, F48A/E50L/R258L, and F48A/E50L/H248N/R258L PNPO forms, so as to evaluate the effect of the variations on the allosteric properties of the enzyme. The initial velocity of PNP conversion into PLP was measured in Na‐HEPES buffer (in which PLP is allowed to accumulate) by varying substrate concentration at different, fixed exogenous PLP concentrations, using 2 μM enzyme. A series of saturation curves were obtained that were used to fit the Michaelis–Menten equation (Figure 5), acquiring apparent *V*_MAX_ and *K*_M_ values as a function of PLP concentration. Both the wild type and the F48A variant show the typical hyperbolic increase of 1/*V*_MAX_ and the parabolic increase of *K*_M_/*V*_MAX_, although this behavior is much less marked with the F48A variant with respect to the wild type enzyme. On the other hand, it is clear that, in the case of both triple and quadruple variants, 1/*V*_MAX_ is not affected by PLP, whereas the *K*_M_/*V*_MAX_ ratio varies linearly with PLP concentration (Figure 5). This behavior, which is typical of a pure competitive inhibition (Cornish‐Bowden, 2012), demonstrates that in these variants PLP is binding only at the active site, so that the general kinetic scheme followed by wild type PNPO reduces to Scheme 1b.

### 2.5 Flexibility of the N‐terminal end

**FIGURE 6:**
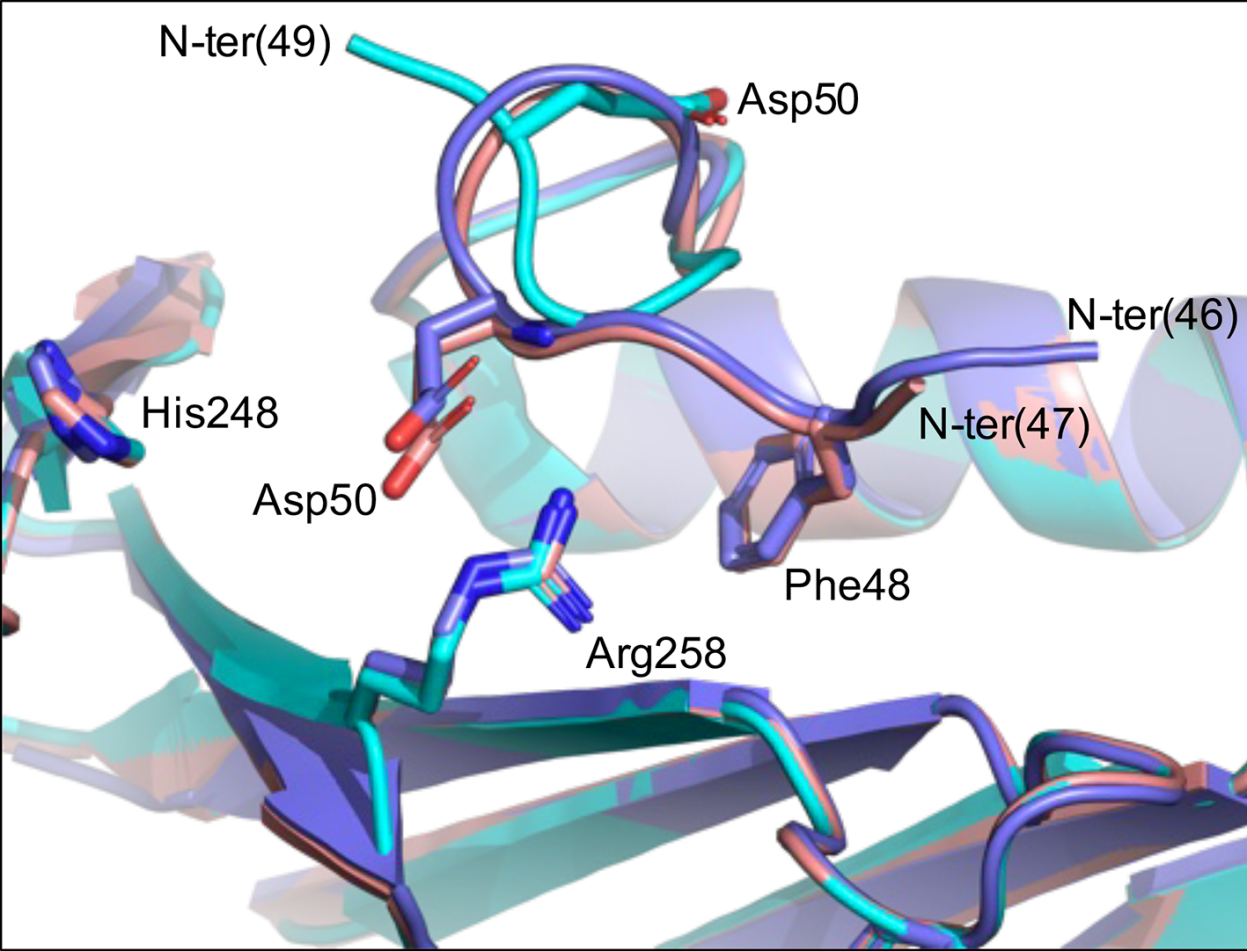
PNPO N‐terminal end. Superimposition of wild type (cyan; PDB: 1NRG), wild type (salmon; this work) and R225H (slate; this work) PNPO forms. The residues involved in PLP binding at the allosteric site are shown in sticks.

**FIGURE 7:**
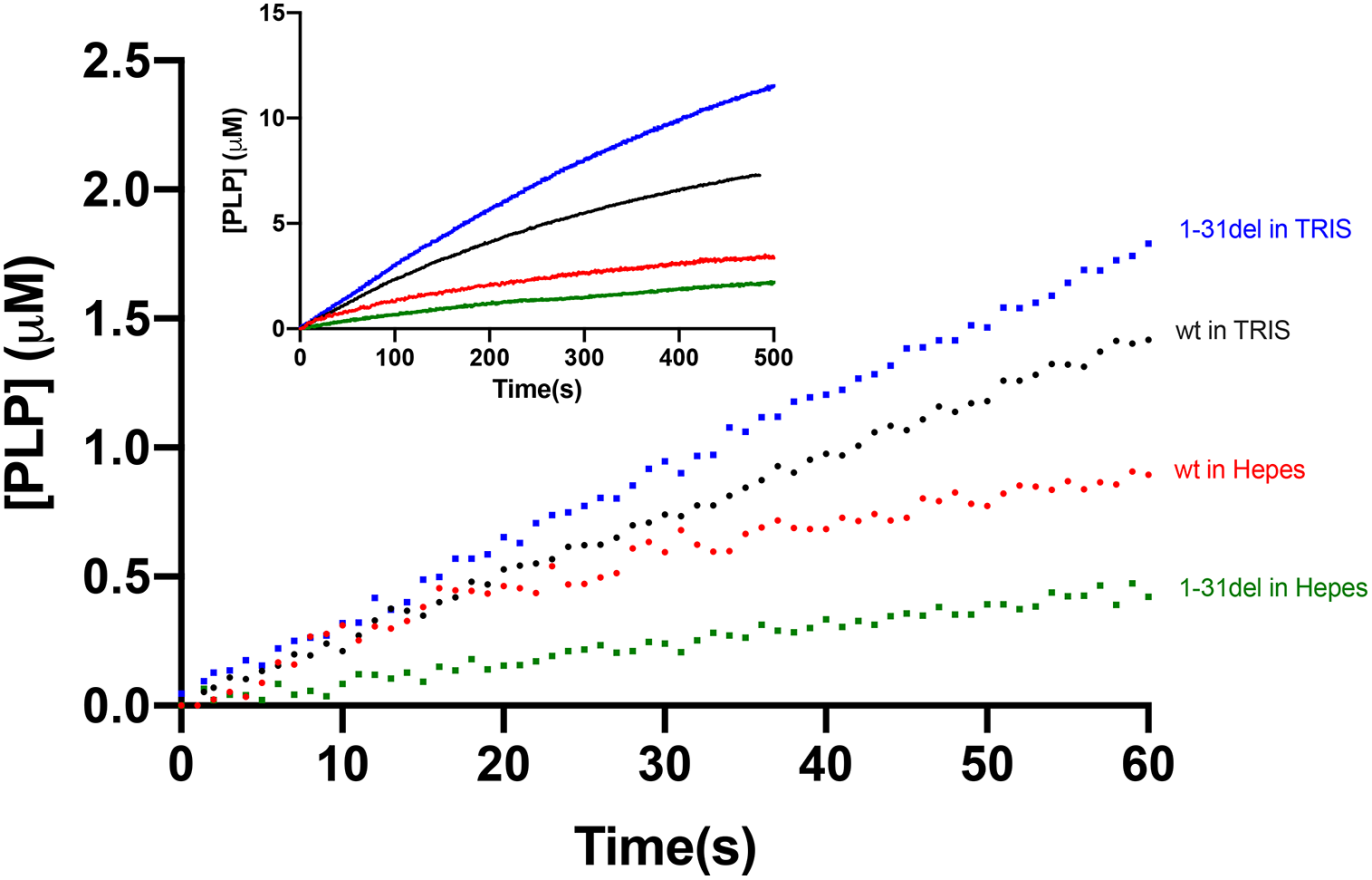
Kinetics of PNP oxidation to PLP in TRIS and HEPES buffers catalyzed by wild type and 1‐31del variant. Kinetics were carried out with 0.5 μM enzyme and 15 μM (wild type) or 35 μM PNP (1‐31del), so as to obtain with both enzymes 90% saturation, in 50 mM TRIS–HCl and 50 mM Na‐HEPES buffers at pH 7.6, containing 5 mM 2‐mercaptoethanol. The inset shows the same graph on a longer time scale.

In order to further confirm the location of the PNPO allosteric site, we made several attempts to obtain the crystal structure of the protein‐PLP complex. With the *E. coli* homologue, any attempt to obtain such a complex with the wild type protein yielded structures in which PLP was bound at the active site, and we could obtain a complex with PLP bound at the allosteric site only when we used a multiple variant form of the enzyme in which the capability to bind ligands at the active site was impaired (Barile et al., 2021). On the basis of this previous experience, we carried out co‐crystallization attempts with either the wild type or the R225H variant of the human protein and PLP. The R225H variation affects a crucial active site residue involved in substrate binding, but is able to bind PLP at the allosteric site and has allosteric properties (Barile et al., 2020). We obtained crystals with both protein forms (Table S1); however, PLP was detected only in the active site of the wild type protein, thus a clear indication about the location of the allosteric site was missing. A substantial part of the N‐terminal end is not visible in these structures: the electron density map starts from Glu46 in the R225H and from Ala47 in the wild type structure. On the other hand, previously published structures of PNPO lack more N‐terminal residues: the first 48 residues in the wild type with PLP at the active site (PDB code: 1NRG; Musayev et al., 2003), the first 50 residues in the R116Q variant (PDB codes: 6H00) and the first 47 residues in the R229W variant (PDB code: 3HY8; Musayev et al., 2009). Moreover, with respect to our structures, the conformation of the N‐terminal end in the previously published structure is different (Figure 6). In 3HY8, Phe48, which is one of the residues crucial in PLP allosteric binding site, is placed in a suitable position to stack the PLP ring at the allosteric site (the same suitable conformation is shown in Figure 1). This feature suggests that a conformational change of the N‐terminal end of the protein could occur when PLP binds at the allosteric site. It is worth noticing that the SDS‐PAGE analysis of the purified recombinant human PNPO reveals the presence of a minor cleaved form of the protein (with an estimated molecular mass of 25.6 ± 0.9 kDa; see Section 4 for details) together with a main form corresponding to the entire protein (estimated molecular mass of 31.0 ± 0.7 kDa) (Figure S5A). Mass spectrometry analyses allowed the identification of the cleavage site. In particular, the analysis of tryptic peptides derived from the lower molecular weight band showed the lack of peptides corresponding to the N‐terminal end of the protein. The analysis of purified PNPO in solution by MALDI‐TOF confirmed the lack of the N‐terminal portion of the protein and the detected mass value, with an accuracy of 0.06%, suggested that the PNPO form corresponding to the lower molecular weight band starts from Arg38 (Figure S6). The proteolytic cleavage of PNPO is not caused by the action of proteases during the purification, but it takes place in the bacterial cells, as evident from the western blot analysis of cell extracts (Figure S5B). This observation indicates a marked flexibility of the N‐terminus, and could explain the fact that this region is missing in the electron density map of all the crystal structures solved so far. Since the N‐terminal region of PNPO is involved in its allosteric properties, we decided to produce and characterize a deleted form of the protein lacking the first 31 residues (starting from Met32). The recombinant deleted variant (1‐31del) purifies as a single protein form, with the expected molecular weight (27.7 ± 0.1 kDa) (Figure S5A), suggesting that the cleavage at Arg38 observed in wild type PNPO is not taking place, probably because of a reduced flexibility of the N‐terminal region in this variant. The far‐UV CD spectrum of the 1‐31del variant is mostly superimposable with respect to that of the wild‐type, except in the 225–240 nm region where the Cotton effect is more pronounced, possibly as a consequence of the lack of the N‐terminal region (Figure S4). The kinetic parameters of the deleted variant are different from those of the wild type PNPO (Table 1), with a threefold higher K_M_ and a sevenfold higher k_CAT_. The most interesting features of the 1‐31del form are a lower K_D_ for PLP binding (threefold lower with respect to wild type PNPO), which indicates a higher affinity for the allosteric site, and the related more marked allosteric inhibition, which is evident from the comparison of the reaction kinetics measured in TRIS and HEPES buffers (Figure 7). The lack of linearity in the early phase of reaction kinetics carried out in HEPES prevented a correct measurement of the initial velocity and therefore a detailed characterization of PLP inhibition as done with the other PNPO variant forms.

## 3 DISCUSSION

Pyridoxal 5′‐phosphate is a crucial cofactor in biochemistry, known for its high versatility as a catalyst, enabling the proper function of numerous enzymes in a wide range of metabolic processes (Percudani & Peracchi, 2003). However, it is worth noticing that PLP is among the top 30 metabolites that are particularly susceptible to damage (Salazar et al., 2016), and it is a highly reactive compound. Maintaining PLP homeostasis inside the cell is vital for proper cellular functions. Several mechanisms contribute to this regulation, and one interesting aspect is how PLP itself plays a role in controlling its production through an allosteric interaction with PNPO. When PLP levels are high, PLP binds to an allosteric site on PNPO, which inhibits the enzyme activity, thus controlling the rate of its own synthesis. It is interesting to notice that the ability of PLP to allosterically regulate the activity of the PNPO enzyme appears to be a conserved feature across different species, including both *E. coli* and humans (Barile et al., 2019, 2020). This suggests that this regulatory mechanism is of fundamental importance in maintaining PLP homeostasis in various organisms. It is expected that a structural conservation in the location of the allosteric site on the enzyme across different species would exist. In this work, we have demonstrated that in human PNPO the PLP allosteric site is located in the same area of the dimeric protein structure with respect to the *E. coli* homologue, although the amino acid residues involved in the interaction with PLP are completely different. In the *E. coli* enzyme, we have previously shown that an arginine cage retains PLP at the interface between the enzyme subunits (Barile et al., 2021). Interestingly, in the allosteric site of human PNPO, PLP makes interactions with amino acid residues that are similar to those established in the active site of PLP‐dependent enzymes, that is, the double‐stacking between aromatic residues, the interaction between an acidic residue (glutamate or aspartate) and the N‐atom of the pyridine ring, as well as a stabilizing interaction of the phosphate group with an arginine residue (Figure 1). Although our x‐ray crystallography analyses were not successful in identifying the location of the PLP allosteric site, the results obtained with the site‐directed variants clearly show that the amino acid residues identified in the docking analysis are directly involved in the allosteric properties of the enzyme. In particular, the variant affecting four of the residues located in the allosteric site (F48A, E50L, H248N and R258L) is competitively inhibited by PLP, showing that in this enzyme form PLP can only bind at the active site (Scheme 1, Figure 5). Actually, the single F48A variation is sufficient to drastically alter the allosteric properties of PNPO, highlighting the crucial importance of the N‐terminal region and of the stacking interaction established by this residue with PLP. Our crystallographic analyses and the results concerning the proteolysis of PNPO N‐terminal region indicate that this region of the protein is very flexible. The deletion of the first 31 N‐terminal residues yields a form of the enzyme (the 1‐31del variant) that maintains all its functional features but shows a more marked allosteric behavior. This observation suggests that the N‐terminal region of the protein is not directly involved in ligand binding and catalysis but influences the conformational equilibrium of the protein and its allosteric properties. The flexibility of the N‐terminal region is a common feature of PNPO from different sources. The purified recombinant *E. coli* PNPO yields electron density maps that are missing the first 4–19 residues (PDB: 1JNW; 1G76; 1G77; 1G78; 1G79; 1WV4; 1DNL; 6YLZ; 6YMH). Structures of PNPO from other sources are also missing the first 24–25 residues in the electron density map, such as *Mycobacterium tuberculosis* (PDB: 2A2J) and *Saccharomyces cerevisiae* (PDB: 1CI0). It appears that, although the PLP allosteric sites of the human and *E. coli* PNPOs are made of completely different residues, the allosteric properties and structural features of the two homologue proteins are very similar to each other. This conclusion is supported by the results obtained from the characterization of the R161C pathogenic variant of the human enzyme (Barile et al., 2020). The R161 residue is located at the active site and interacts with the phosphate moiety of the substrate vitamers. Although the altered kinetic parameters of this variant are sufficient to explain its pathogenic character, the complete lack of allosteric properties of the R161C variant is what really strikes our attention in view of its connection with the observations made with the *E. coli* PNPO. In fact, the corresponding conserved arginine residue in *E. coli* PNPO (Arg133) is located in a protein domain involved in the allosteric coupling between the active site and the PLP allosteric site (Barile et al., 2021). PLP binding at the allosteric site of *E. coli* PNPO results in the disorder of the large structural region made by residues 123–166 and also uncoils a short helix (residues 192–200), which contains Arg197 (corresponding to Arg225 of human PNPO) that plays a crucial role in binding the vitamer substrates at the active site.

Given the importance of PNPO as a target for the action of chemotherapeutic agents (Chen et al., 2017; Ren et al., 2019; Zhang et al., 2017), the identification of the PLP allosteric site will offer in the next future a valuable opportunity to develop PNPO inhibitors that effectively and specifically bind at the allosteric site.

## 4 METHODS

### 4.1 Bioinformatics analysis and docking

Orthologous sequences of PNPO were found using Blast and aligned using (Larkin et al., 2007) for MSA and further refined through manual inspection to enhance alignment quality. Using these refined alignments, the CAMPO (Paiardini et al., 2005) tool was employed to evaluate the extent of evolutionary conservation for individual protein residues and to map the evolutionary conservation on the structure of human PNPO (PDB: 1NRG; Zhang et al., 2017). Molegro Virtual Docker ver. 5.5 (MVD; Thomsen & Christensen, 2006) was used to dock PLP into the identified clefts, using default settings and different human structures to mimic the flexibility of the N‐terminal region (PDB codes: 1NRG, Musayev et al., 2003; 6H00; 3HY8, Musayev et al., 2009).

### 4.2 Site‐directed mutagenesis, expression, and purification of PNPO variant enzymes

Site‐directed mutagenesis was performed by QuickChange methodology (Agilent). Plasmid expressing the F48A variant was obtained using as template the pET28‐*PNPO* plasmid containing the cDNA coding for wild type human PNPO, cloned into the expression vector pET28a(+) (Musayev et al., 2003). The other plasmids carrying the mutations were obtained as indicated in Table S2, which also reports the sequence of the mutagenic primers, synthesized by Metabion International AG (Steinkirchen, Germany). The 1‐31del variant was also made by QuickChange methodology through the generation of a mutant form carrying an additional *Nde*I restriction site encompassing Met32 (see Table S2 for mutagenic primer sequence). The deletion was then performed by *Nde*I digestion of the mutant plasmid, followed by agarose gel purification of the plasmid linear form and subsequent recircularization by T4 DNA ligation. All mutants were fully sequenced at Microsynth Seqlab (Göttingen, Germany). Competent *E. coli* BL21(DE3) cells (Novagen) were transformed with pET28‐*PNPO* constructs carrying all the variations. Purification of the variants was carried out as described for the wild type in Barile et al. (2020).

### 4.3 Electrophoresis and Western blot analyses

The molecular weight of purified PNPO samples analyzed by SDS‐PAGE was estimated by comparison with protein standards using the MS Image Capture‐A (Major Science, Taiwan) software. For the immunological detection of recombinant PNPO in cell lysates, aliquots of the bacterial cultures expressing either wild‐type or 1‐31del PNPO forms were withdrawn after 6 h from the addition of IPTG and centrifuged. Cell pellets were suspended in 250 μL of 20 mM potassium phosphate buffer, pH 7.6, and sonicated for 2 min with a 20‐s ON + 20‐s OFF cycle. SDS‐PAGE was performed on 20 μg protein samples, as estimated with a Bradford assay performed on lysates. Western blotting analyses were performed with a Bio‐Rad mini trans‐blot® system (BioRad, Hercules, CA, USA). The membrane was blocked for 1 h with the AdvanBlock™‐Chemi blocking solution (Advansta Inc., USA), incubated overnight at 4°C with an anti‐PNPO rabbit pAb (ABclonal, USA) diluted 1:1000 in TBST solution and then with a goat anti‐rabbit IgG (h/l chain) HRP‐conjugated secondary antibody (Bethyl Laboratories Inc., USA), at room temperature. Chemiluminescence was detected with a ChemiDoc™ Bio‐Rad Imaging system, after incubating the membrane with Pierce™ ECL (Thermo scientific Inc., USA).

### 4.4 Production of the apo‐form of PNPO

The apo‐enzyme forms were prepared at low pH through a phenyl sepharose chromatographic step (Cytiva), as previously described (Musayev et al., 2003).

### 4.5 Spectroscopic measurements

All absorption spectra were acquired in 20 mM potassium phosphate pH 7.6 at 25°C. UV–visible spectra were recorded using a Hewlett‐Packard 8453 diode‐array spectrophotometer. Far‐UV (190–250 nm) CD spectra were acquired on a Jasco 710 spectropolarimeter equipped with a DP 520 processor employing 0.1‐cm path length quartz cuvettes.

### 4.6 Kinetic studies

PNP was obtained from PLP (98% pure; Merck) according to the method described by Argoudelis (1986). PMP was synthesized by reductive amination of PLP, by adapting the procedure for PNP described by Argoudelis. Briefly, pure PLP (530 mg) (*Merck*) was dissolved in 8 mL of a 15% aqueous ammonia solution. The primary aldimine was reduced to the amine by slowly adding sodium borohydride (50 mg) to the solution, which decolorized within 1 h. The spectral change was monitored by UV–Vis spectroscopy to verify the completeness of the reduction (as indicated by the disappearance of the absorption peak at 388 nm). PMP solution was further alkalized to a pH of 13 by adding NaOH. The reaction mixture was further purified by ion exchange chromatography as described by Argoudelis, and concentrated under vacuum at 40°C in a rotary evaporator, until turbidity was visible. The crystals that formed were collected by paper filtration, then washed and desiccated. Identity and purity of the final product were assessed by reverse‐phase HPLC and UV–Vis spectroscopy.

As reported in Barile et al. (2020), activity assays were carried out in 50 mM Tris–HCl pH 7.6, containing 5 mM 2‐mercaptoethanol, at 37°C. The progress of the reaction was followed at 414 nm, where the characteristic aldimine product PLP‐Tris absorbs maximally with a molar absorbance coefficient of 4253 M^−1^ cm^−1^. Kinetic constant measurements were performed using 2 μM of each PNPO variants, and varying PNP or PMP concentrations from 0.5 to 800 μM. The values of *K*_M_ and *k*_CAT_ were determined from least‐squares fitting of initial velocity data as a function of substrate concentration to a quadratic Equation (1), in which *v*_*i*_ is the initial velocity of the reaction, *k*_CAT_ [*E*_0_] corresponds to *V*_MAX_, the maximum velocity of the reaction, [PNP_0_] is the total substrate concentration, [*E*_0_] is the total enzyme concentration and *K*_D_ is the dissociation constant of the substrate binding equilibrium that, assuming a rapid establishment of the equilibrium, is equivalent to *K*_M_.

(1) $${\text{v}}_{\text{i}}=k_{CAT}\left[E_{0}\right]\frac{\left[PNPO_{0}\right]\;+\left[E_{0}\right]+K_{D\;-}\sqrt{{\left(\left[PNPO_{0}\right]+\left[E_{0}\right]+K_{D}\right)}^{2}-4\left[PNPO_{0}\right]\left[E_{0}\right]}}{2\;\left[E_{0}\right]}$$

Inhibition kinetic measurements were carried out in 50 mM Na‐HEPES pH 7.6, containing 5 mM 2‐mercaptoethanol. In this case, product formation was followed at 388 nm, where PLP absorbs maximally with a molar absorbance coefficient of 5330 M^−1^ cm^−1^. All saturation curves shown in Figure 5, obtained by varying PNP at a fixed PLP concentration, were independently analyzed using the Michaelis–Menten equation, obtaining apparent *V*_MAX_ and apparent *K*_M_ values, which were then used to calculate 1/*V*_MAX_ and *K*_M_/*V*_MAX_. Fitting of 1/*V*_MAX_ and *K*_M_/*V*_MAX_ was carried out using an increasing hyperbolic equation and a parabolic equation, respectively.

### 4.7 Analysis of PLP binding equilibrium

Analyses of PLP binding, based on FMN fluorescence increase observed upon binding of PLP to PNPO, were performed as described in Barile et al. (2020). PLP binding to apo‐forms of wild type and variant PNPO was analyzed owing to the protein intrinsic fluorescence emission quenching observed upon binding under excitation at 280 nm. Emission spectra were recorded from 300 to 450 nm, with excitation and emission slits set at 3 and 5 nm, respectively. Fluorescence emission values between 335 and 345 nm were averaged and analyzed according to Equation (2).

(2) $$\mathrm{Fractional}\;\mathrm{variation}=\begin{array}{l} F_{\max}\;-(F_{\max\;}\times \frac{\left[\mathrm{PLP}\right]+\left[E\right]+K_{D}-\sqrt{{\left(\left[\mathrm{PLP}\right]+\left[E\right]+K_{D}\right)}^{2}-4\;[\mathrm{PLP}]\;[E]}}{2\;\left[E\right]}) \end{array}$$

where [PLP] is the PLP concentration, [*E*] is the total enzyme concentration, *K*_D_ is the value of the dissociation constant, and *F*_max_ is the observed total fluorescence change.

Concerning PLP binding to PNPO revealed by DSF, experiments were carried out in 50 mM Na‐HEPES buffer pH 7.6, using 2 μM of each PNPO variants on Real Time PCR Instrument (CFX Connect Real Time PCR system, Bio‐Rad) as described in Barile et al. (2020).

### 4.8 Data analysis

Data were analyzed using the software Prism (GraphPad Software Inc., San Diego, CA). Illustrations were created with BioRender.com.

### 4.9 Protein crystallization, data collection, and structure solution

After purification, wild type PNPO and the R225H variant were concentrated to a final concentration of ~9 and 5 mg/mL, respectively. The solutions contained 20 mM K_3_PO_4_ (pH 7.8), 150 mM NaCl, and 5 mM 2‐mercaptoethanol. PLP was added in excess and the solutions were centrifuged for 10 min at 16,000×*g* at 4°C to remove protein aggregates. Automated crystallization screenings were performed at 20°C with a Crystal Phoenix crystallization robot (Art Robbins) by the sitting drop vapor diffusion method using a 1:1 ratio of protein and reservoir solutions. For the wild type PNPO, PLP was added to the protein solution in a 1:18 molar ratio (protein:PLP). Well‐diffracting crystals were obtained at 20°C with a mother liquor containing 12% v/v PEG 6000, 0.1 M sodium citrate pH 5.6, and 0.1 M lithium sulfate. For R225H variant, PLP was added in a 1:15 molar ratio (protein:PLP). The best conditions identified by automated screening were optimized manually by the hanging drop method. Crystals were obtained at 20°C with a mother liquor containing 1 M (NH_4_)_2_SO_4_, 2% PEG400 in 0.1 M HEPES pH 7.5. To further promote the binding of PLP to the allosteric site, additional soaking of crystals in PLP solution (1:15 protein:PLP ratio) was performed. Crystals were fished, mounted on nylon loops, and cryoprotected by immersion in a solution containing 80% (v/v) mother liquor and 20% (v/v) glycerol, before flash‐freezing in liquid nitrogen. Single wavelength datasets were collected at the beamline XRD2 at the Elettra‐Sincrotrone of Trieste using a Dectris Pilatus 6M detector at a temperature of 100°K. Both datasets were processed and scaled with XDS (Kabsch, 2010) and AIMLESS (Evans & Murshudov, 2013). Crystal parameters and collection statistics are available in Table S1. The structures were solved by molecular replacement with MOLREP (Vagin & Teplyakov, 2010) using 1NRG as search model. Structures refinement was performed with the program REFMAC5 (Murshudov et al., 2011), whereas models were built in COOT (Emsley et al., 2010).

### 4.10 Mass spectrometry analyses

SDS‐PAGE protein bands of interest were excised and subjected to trypsin proteolysis. Briefly, after some washing steps (50 mM ammonium bicarbonate with or without either 50% or 100% acetonitrile), the bands were reduced (10 mM DTT) and alkylated (55 mM IAA). An amount of 100 ng of trypsin (Trypsin Gold, Mass Spectrometry Grade, Promega) in 25 mM of ammonium bicarbonate solution was added to the bands and let to incubate at 37°C for 3 h. The peptide mixtures were analyzed on a MALDI‐TOF mass spectrometer, ultrafleXtreme (Bruker, Bremen DE, Germany), equipped with a smartbeam‐II laser. An aliquot (1 mL) of each mixture was mixed with an equal volume of α‐cyano‐4‐hydroxycinnamic acid matrix, solubilized in an aqueous solution of acetonitrile (70%) and trifluoroacetic acid (0.1%). The acquisition of spectra was in reflector and positive mode. Concerning the mass spectrometry analysis of purified PNPO in solution, an aliquot (1 mL) of the protein solution was analyzed by mass spectrometry, after mixing with sinapic acid, solubilized in an aqueous solution of acetonitrile (70%) and trifluoroacetic acid (0.1%), in different ratio. The acquisition was in linear and positive mode. The analysis of all acquired spectra was achieved by Flex Analysis software.

## AUTHOR CONTRIBUTIONS

**Anna Barile:** Investigation; methodology. **Claudio Graziani:** Investigation; methodology; visualization. **Lorenzo Antonelli:** Investigation. **Alessia Parroni:** Investigation. **Annarita Fiorillo:** Investigation; visualization; writing – original draft; writing – review and editing. **Martino Luigi di Salvo:** Investigation; writing – review and editing. **Andrea Ilari:** Investigation; methodology; writing – original draft. **Alessandra Giorgi:** Investigation; methodology; writing – original draft. **Serena Rosignoli:** Investigation. **Alessandro Paiardini:** Investigation; methodology; writing – original draft; writing – review and editing. **Roberto Contestabile:** Conceptualization; writing – original draft; writing – review and editing; funding acquisition; supervision; project administration; validation; data curation; resources; visualization. **Angela Tramonti:** Conceptualization; investigation; funding acquisition; writing – original draft; writing – review and editing; validation; project administration; supervision; methodology; data curation; resources; visualization.

## CONFLICT OF INTEREST STATEMENT

The authors declare that they have no conflicts of interest with the contents of this article.

## Supporting information


**Figure S1.** Schematic approach for the identification of the allosteric site of PLP in human PNPO.
**Figure S2.** Saturation curves obtained with PNPO variants in TRIS buffer using PNP as substrate.
**Figure S3.** Saturation curves obtained with PNPO variants in TRIS buffer using PMP as substrate.
**Figure S4.** Far‐UV CD spectra of the indicated PNPO variants.
**Figure S5.** Gel electrophoresis and western blot analysis.
**Figure S6.** Mass spectrometry analysis.
**Table S1.** Crystallographic data and refinement statistics.
**Table S2.** Oligonucleotide primers used in this study.
