# Supplementary material for: Identification of the pyridoxal 5′‐phosphate allosteric site in human pyridox(am)ine 5′‐phosphate oxidase
Source: Protein Sci. 2024 Jan 23;33(2):e4900. doi: 10.1002/pro.4900 (PMC10804683; doi:10.1002/pro.4900)
Supplement: Supplementary file 1 — Figure S1. Schematic approach for the identification of the allosteric site of PLP in human PNPO. Figure S2. Saturation curves obtained with PNPO variants in TRIS buffer using PNP as substrate. Figure S3. Saturation curves obtained with PNPO variants in TRIS buffer using PMP as substrate. Figure S4. Far‐UV CD spectra of the indicated PNPO variants. Figure S5. Gel electrophoresis and western blot analysis. Figure S6. Mass spectrometry analysis. Table S1. Crystallographic data and refinement statistics. Table S2. Oligonucleotide primers used in this study. [file PRO-33-e4900-s001.pdf]

Fig. S1

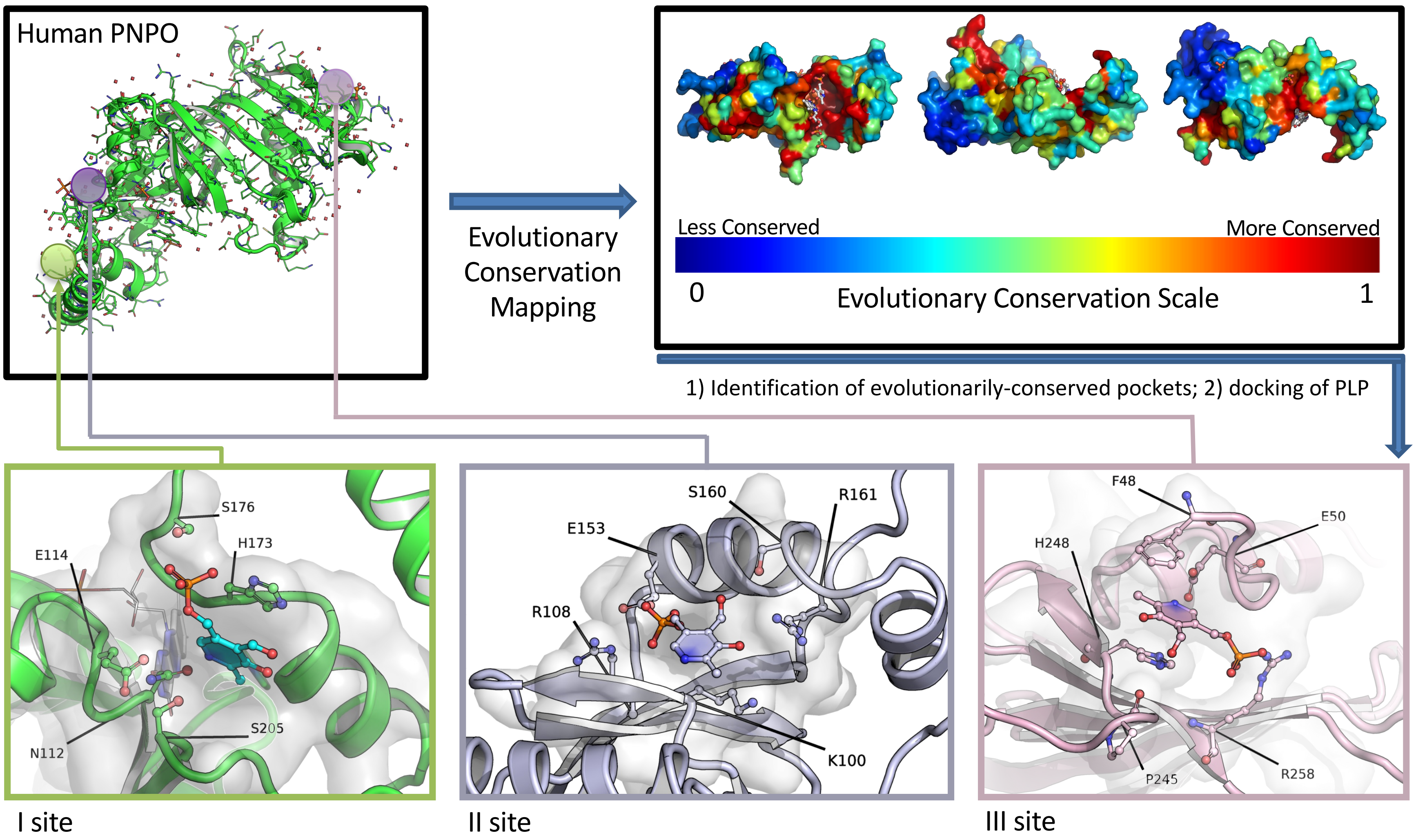

Schematic approach for the identification of the allosteric site of PLP in human PNPO. To identify surface pockets within the human PNPO protein structure potentially binding PLP, we analysed evolutionarily conserved residues of orthologous PNPO enzymes and mapped this conservation on its surface; then, we conducted PLP docking into these recognized pockets to evaluate its potential binding orientation. The docking investigations revealed the presence of three plausible binding sites, whose position is reported on the monomeric polypeptide chain.

Fig. S2

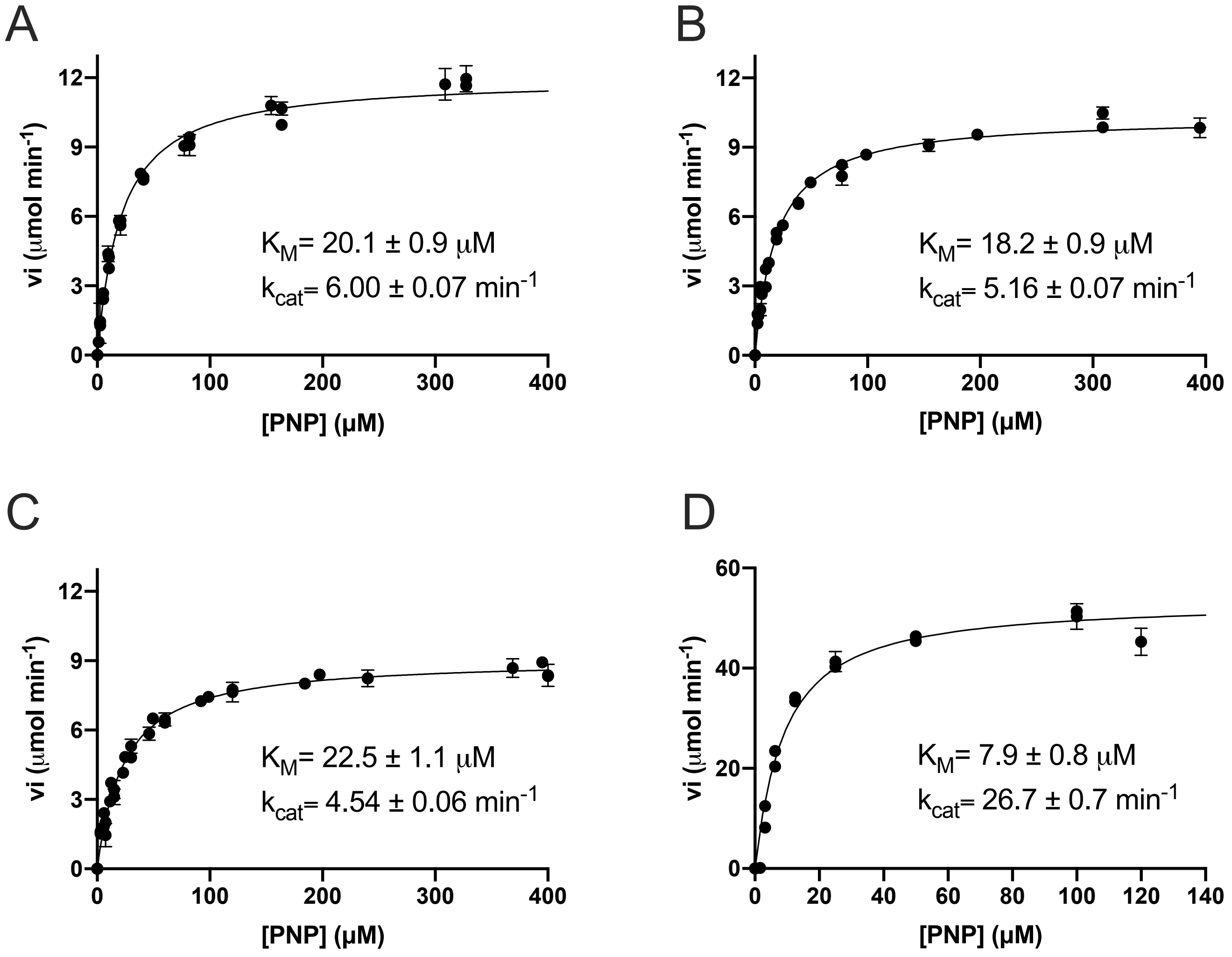

Saturation curves obtained with PNPO variants in TRIS buffer using PNP as substrate. Initial velocity of the reaction catalysed by the F48A (A), F48A/E50L/R258L (B), F48A/E50L/H248N/R258L (C), and 1-31del PNPO variants as a function of PNP concentration. These saturation curves were analysed using the quadratic equation 1, obtaining the kinetic parameters indicated both in the figure and in the Table 1.

Fig. S3

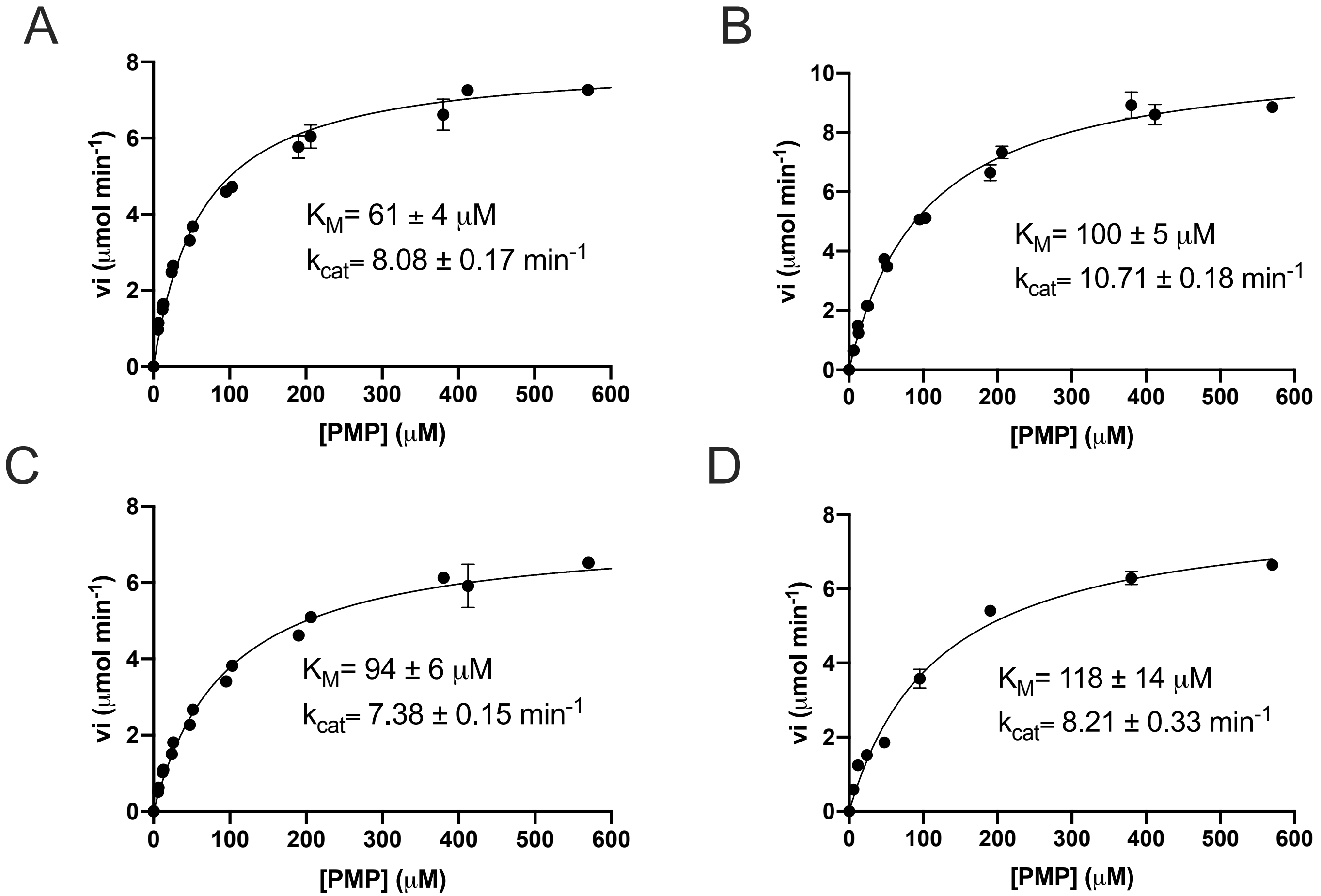

Saturation curves obtained with PNPO variants in TRIS buffer using PMP as substrate. Initial velocity of the reaction catalysed by the wild type (A), and the F48A (B), F48A/E50L/R258L (C) and F48A/E50L/H248N/R258L (D) PNPO variants as a function of PMP concentration. These saturation curves were analysed using the quadratic equation 1, obtaining the kinetic parameters indicated both in the figure and in the Table 1.

Fig. S4

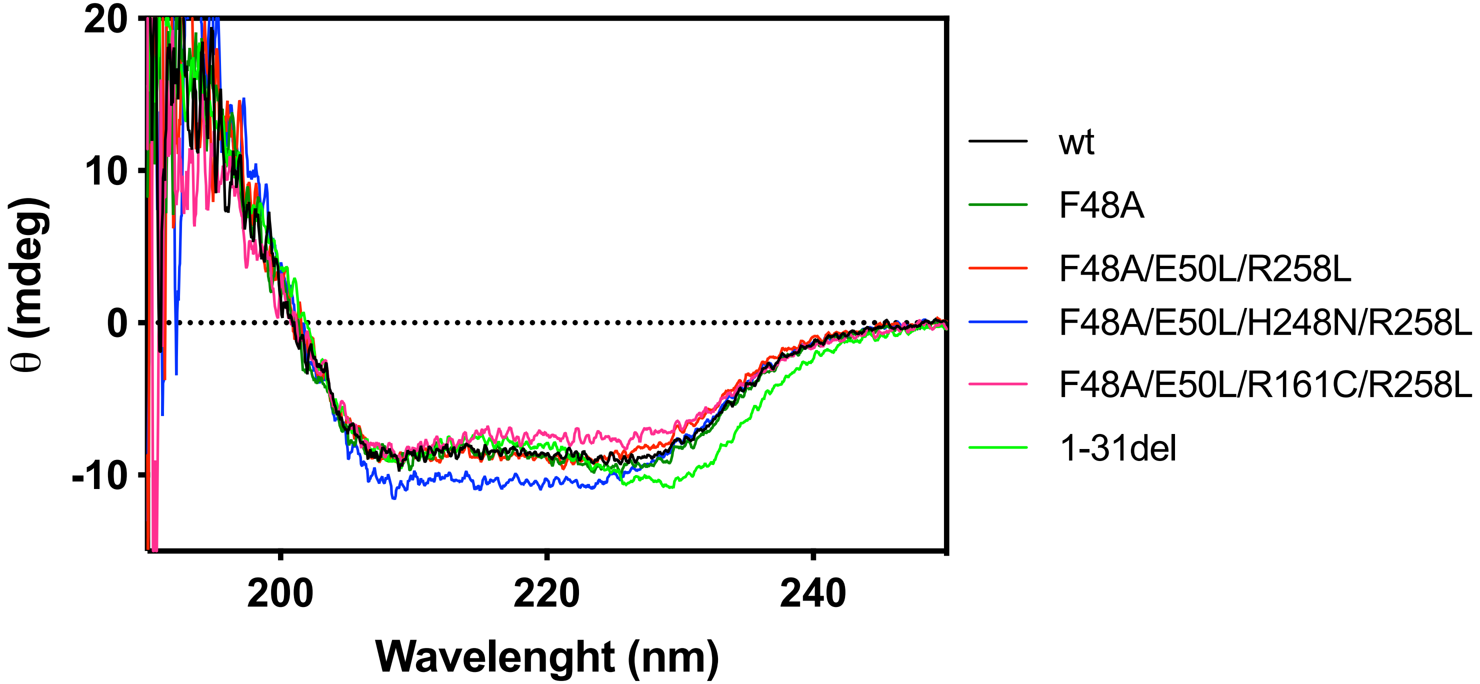

Far-UV CD spectra of the indicated PNPO variants. All spectra were measured in 20 mM potassium-phosphate buffer, pH 7.6.

Fig. S5

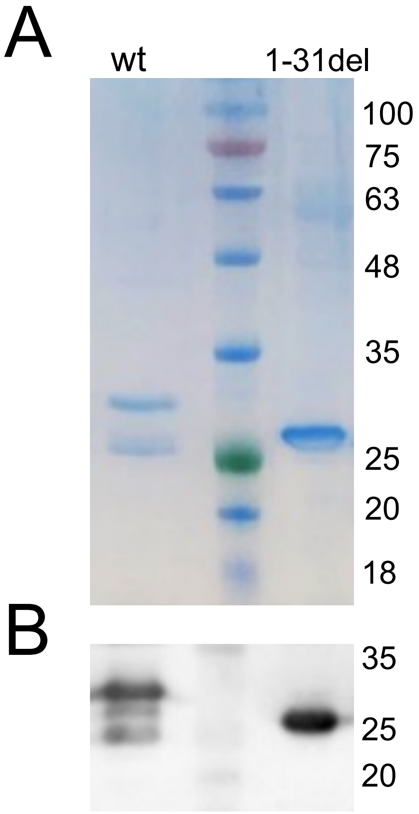

Gel electrophoresis and western blot analysis. A) SDS-PAGE analysis of the purified recombinant wt and 1-32del PNPO forms. B) Western blot analysis of the total extract from *E. coli* BL21 containing the pET28-*PNPO* plasmid.

Fig. S6

```
MTCWLRGVTA TFGRPAEWPG YLSHLCGRSA AMDLGPMRKS YRGDREAFEE THLTSLDPVK
QFAAWFEEAV QCPDIGEANA MCLATCTRDG KPSARMLLLK GFGKDGFRFF TNFESRKGKE
LDSNPFASLV FYWEPLNRQV RVEGPVKKLP EEEAECYFHS RPKSSQIGAV VSHQSSVIPD
REYLRKKNEE LEQLYQDQEV PKPKSWGGYV LYPQVMEFWQ GQTNRLHDRI VFRRGLPTGD
SPLGPMTHRG EEDWLYERLA P
```

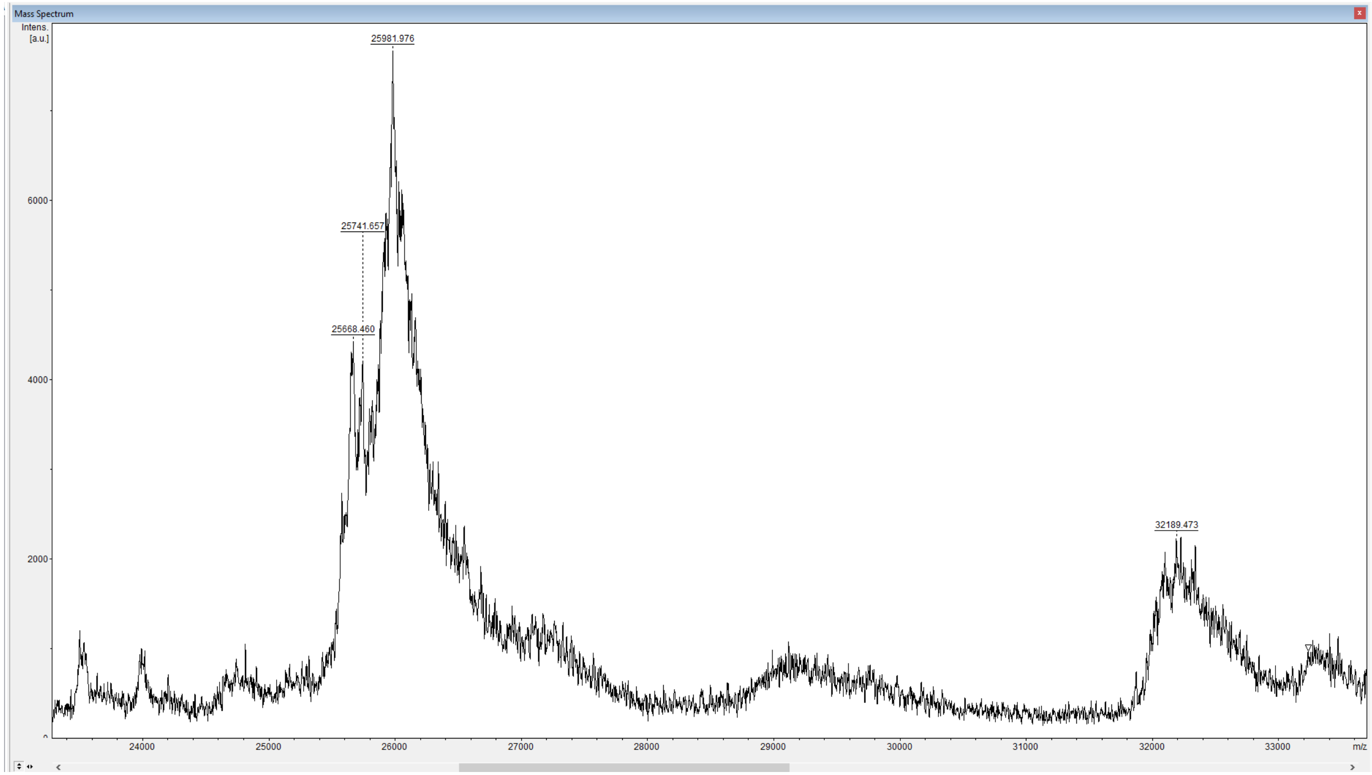

Mass spectrometry analysis. The MALDI ToF spectrum of PNPO in solution reveals that the major form at 25.981,97 Da should correspond to PNPO form starting from Arg38 (highlighted in red), as expected from theorethical mass 25965.29±0.06% by amino acid sequence.

Table S1. Crystallographic data and refinement statistics

	hPNPO WT	*hPNPO R225H*
PDB entry:	8QYT	8QYW
Number of images:	720	720
Space group:	$P3_121$	$P3_121$
Unit cell dimensions (Å):	82.38 82.38 59.14	82.88 82.88 60.64
Resolution range (Å):	45.576-1.691 (1.720-1.691)	41.440-2.747 (2.794-2.747)
No of reflections:	484689 (24805)	119301 (6377)
No of unique reflections:	26324 (1316)	6519 (323)
Completeness (%):	100.0 (100.0)	99.8 (100.0)
I/sigma (I):	30.2 (2.2)	21.5 (2.4)
CC ½(%):	100.0 (83.9)	99.9 (86.6)
Mosaicity (°):	0.207	0.200
**Refinement**		
Resolution range (Å):	45.6-1.69	41.5-2.75
Wilson B-factors (Å^2)	30.9	79.6
Rwork:	0.17	0.198
Rfree:	0.22	0.239
Used Reflections:	26324	6519
**Number of atoms**		
Protein:	1806	1709
Solvent:	138	4
Cofactor:	31	31
PLP:	16	
BME:	4	
PO_4^{3-}		15
**Mean B factor (Å^2)**		
Protein:	35.927	81.975
Solvent:	44.617	76.460
Cofactor:	24.908	66.550
PLP:	40.801	
BME	52.585	
PO_4^{3-}		81.581
Rmsd bond length (Å):	0.0111	0.0018
Rmsd angles (°):	1.6643	0.7273
**Ramachandran plot statistics**		
N° res. favored (%):	200 (97.6)	194 (95.1)
N° res. allowed (%):	5 (2.4)	10 (4.9)

Table S2. Oligonucleotide primers used in this study.

**Variant**	**Template**	**primers**
F48A	pET28-*PNPO*	for: GACCGAGAGGCAGCGGAGGAGACTCATC<br>rev: GATGAGTCTCCTCCGCTGCCTCTCGGTC
F48A/E50L	pET28-*PNPOF48A*	for: GACCGAGAGGCAGCGGAGCTGACTCATC<br>rev: GATGAGTCAGCTCCGCTGCCTCTCGGTC
F48A/E50L/R258L	pET28-*PNPOF48A/E50L*	for: GGCTCTATGACTTACTTGCACCTTAAG<br>rev: CTTAAGGTGCAAGTAACTCATAGAGCC
F48A/E50L/H248N/R258L	pET28-*PNPO*F48A/E50L/R258L	for: GGCCCATGACCAACCGCGGGGAG<br>rev: CTCCCCGCGGTTGGTCATGGGCC
F48A/E50L/R161C/R258L	pET28-*PNPO*F48A/E50L/R258L	for: CTACTTCCACTCCTGCCCCAAGAGCAG<br>rev: CTGCTCTTGGGGCAGGAGTGGAAGTAG
1-32del	pET28-*PNPO*	for: GTCGCAGTGCTGCATATGGACCTGGGAC<br>rev: GTCCCAGGTCCATATGCAGCACTGCGAC
